# Associations between MDMA/ecstasy, classic psychedelics, and suicidal thoughts and behaviors in a sample of U.S. adolescents

**DOI:** 10.1038/s41598-022-25658-5

**Published:** 2022-12-19

**Authors:** Grant Jones, Diego Arias, Matthew Nock

**Affiliations:** Department of Psychology, Harvard University, 33 Kirkland St, Cambridge, MA 02138 USA

**Keywords:** Psychology, Human behaviour

## Abstract

Suicide is one of the leading causes of death amongst adolescents and decades of research have failed to curb suicide rates within this population. There is thus a need to better understand factors that correlate with adolescent suicidal thoughts and behaviors (STBs). MDMA/ecstasy and classic psychedelics represent two areas for exploration, as use of these substances has been associated with both increased and lowered odds of STBs. Thus, the goal of this study was to test the associations between MDMA/ecstasy and classic psychedelics (psilocybin, peyote, mescaline, LSD) and STBs in a nationally representative sample of U.S. adolescents. We tested these associations in a sample of adolescents aged 12–17 years old from the National Survey on Drug Use and Health (2004–2019) (*N* = 262,617) using survey-weighted multivariable logistic regression models. Lifetime psilocybin use was associated with lowered odds of lifetime suicidal thinking, planning, and attempts (aOR range 0.77–0.85). Conversely, LSD was associated with increased odds of these same outcomes (aOR range 1.20–1.35). MDMA/ecstasy, peyote, and mescaline did not share associations with STBs. Our study demonstrates that individual classic psychedelics share varying relationships to STBs among adolescents. Future cross-sectional and longitudinal studies are needed to further elucidate the link between classic psychedelic use and STBs in youth.

## Introduction

Suicide is the second leading cause of death among adolescents, and suicide rates in this population continue to rise despite years of research and medical advances^1,2^. Even with a nearly exponential increase in the number of RCTs studying suicidal thoughts and behaviors (STBs) in the last decades, existing treatments have continued to have limited efficacy^3,4^. Moreover, given that current research has not yielded improvements in treatment efficacy nor reductions in STBs among adolescents, there is a need for a wider range of research on factors that correlate with adolescent STBs.

Classic psychedelics (e.g., psilocybin, LSD, peyote, mescaline) and MDMA (3,4-methylenedioxymethamphetamine; also known as “ecstasy”) represent two frontiers for further exploration as use of these substances may share significant associations with STBs and related mental health conditions in adolescents.

Classic psychedelics are a group of naturally-occurring or naturally-derived psychoactive compounds that act as agonists of the serotonin 2A receptor and can give rise to mystical experiences that can in some cases improve one’s perceived quality of life^5,6^. Conversely, these substances also can have negative consequences such as increased anxiety, paranoia, and significant distress associated with acute administration. Some of the most commonly used classic psychedelics are psilocybin (the psychoactive agent in more than 200 species of fungi), lysergic acid diethylamide (LSD; derived from the hydrolysis of an ergot fungus), peyote (a small cactus), and mescaline (the main psychoactive compound in peyote). MDMA, on the other hand, is a synthetic amphetamine derivative that releases serotonin and is known to increase feelings of empathy and social connection; conversely, this substance can also give rise to adverse states of panic, anxiety, and fear as well.

Researchers in the last decade have begun to explore the potential of classic psychedelics as a treatment for a multitude of mental disorders, many of which confer risk for STBs^7,8^. Recent research has shown that classic psychedelics, most namely psilocybin and LSD, can alleviate anxiety and depression (two of the main risk factors of suicide^9,10^). In a 2011 pilot experiment, researchers found that a single session of psilocybin-assisted psychotherapy led to sustained decreases in both anxiety and depression among subjects with advanced-stage cancer^11^. In 2016, these findings were extended in a study that found that improvements in anxiety, depression, quality of life, and optimism due to psilocybin-assisted psychotherapy; furthermore, these improvements continued to be clinically significant at a 6-month follow-up^12^. Similarly, a pilot study in 2014 found that LSD can reduce anxiety when administered in a supervised psychotherapeutic environment^13^. Recent research has also found classic psychedelics to help treat substance use disorders, which are another significant risk factor for suicide^14–16^.

Recent research has also looked at the potential for MDMA to treat PTSD, one of the strongest anxiety-related predictors of suicidal ideation^17^. MDMA-assisted psychotherapy in particular has been found to be not only safe, but also more effective than traditional therapy in patients with treatment-resistant PTSD^18–22^. It is important to note, however, that all of the aforementioned treatment studies on MDMA and classic psychedelics have been conducted using adult samples, underscoring the need for examining the link between these substances and suicidality in adolescents.

Population-based survey studies, while not causal in nature, have helped further establish externally valid links between classic psychedelics and MDMA and STBs; these studies also invite further inquiry into the associations between psychedelics and suicidality in adolescents. Analyzing data from the National Survey on Drug Use and Health (NSDUH), Hendricks et al. (2015a) found that lifetime classic psychedelic use was associated with reduced odds of past-year suicidal thinking, planning, and attempts in a sample of U.S. adults, while the lifetime use of other illicit drugs was associated with increased odds of these same outcomes^23^. A follow-up study by Hendricks et al. (2015b) extended the previous findings to psilocybin specifically and found that it is especially protective with regard to STBs^24^. Additionally, Sexton et al. (2020) found that lifetime classic tryptamine use (e.g. psilocybin and DMT, another classic psychedelic) was associated with decreased odds of psychological distress and suicidal thinking in a sample of U.S. adults as well^25^.

More recently, research by this team has extended previous work by analyzing data from the NSDUH spanning from 2008 to 2019 and found that lifetime MDMA use was associated with reduced odds of past-year suicidal thinking and planning while lifetime psilocybin use was associated with reduced odds of past month psychological distress and past year suicidal thinking in adults^26^. Importantly, this study also found that LSD was associated with *increased* odds of past-year suicidal thinking, indicating that in some cases classic psychedelics can confer increased risk for STBs.

Although previous research has established an association between classic psychedelics and MDMA and STBs, few studies have examined the effects of these substances, let alone their link with STBs, among adolescents. Given that classic psychedelic use among adolescents has been rising^27^ and MDMA is one of the most commonly used illicit substances by adolescents^28^, there is a need to assess the link between these substances and STBs in adolescents.

The aim of this paper is to test the associations between MDMA and four individual classic psychedelics (psilocybin, peyote, mescaline, and LSD) and STBs in adolescents in a nationally representative sample (NSDUH). More specifically, this paper aimed to compare the associations among these substances to those reported in Jones and Nock (2022a)^26^, which found MDMA/ecstasy and psilocybin to confer lowered odds of STBs and LSD to confer increased odds of STBs.

## Method

This study used data from the National Survey on Drug Use and Health (NSDUH; 2004–2019), an annual survey that collects information on substance use and mental health outcomes in a nationally representative sample of the U.S. population aged 12 years and older. We included adolescents aged 12–17 years old from the NSDUH in our analyses (*N* = 262,617). The study was exempt from review from the Harvard IRB as the NSDUH data are publicly available at the following web address (https://www.datafiles.samhsa.gov/). Furthermore, we carried out all methods in this project in accordance with the relevant guidelines and procedures.

### Independent variables

In our study, lifetime use of MDMA/ecstasy and four commonly used classic psychedelics (psilocybin, peyote, mescaline, LSD) served as independent variables. These independent variables were selected as this study aimed to extend findings from Jones and Nock (2022a), which assessed the relationships of these substances to STBs in a sample of U.S. adults.

### Covariates

In line with Jones and Nock (2022a), our study included the following demographic factors and substance use variables as a priori covariates in our analyses: sex, age, income, race, education, self-reported engagement in risky behavior, and lifetime use of the following substances (cocaine, heroin, PCP, inhalants, pain relievers, tranquilizers, stimulants, sedatives, marijuana). Furthermore, these covariates also match those from numerous population-based psychedelic studies as well^23,25,29–34^.

### Dependent variables

We used three binary suicide-related variables (yes/no) as outcomes in our study: lifetime suicidal thinking, lifetime suicidal planning, and a lifetime suicide attempt. Our time horizons for the dependent variables in this study deviate from Jones and Nock (2022a), which examined the associations between psychedelic use and past year STB outcomes for adults; however, all adolescent suicidality variables in the NSDUH assess lifetime STBs, thus explaining our approach for the current study.

### Analyses

We used survey-weighted multivariable logistic regression to estimate the associations between our independent variables and our STB outcomes in this study. For all analyses, we used the ‘Survey’ package in R version 4.1.2 as this software allowed us to incorporate the complex design and survey-weighting from the NSDUH into our analyses.

## Results

The demographics of our sample are presented in Table 1, stratified by those who have versus have not tried classic psychedelics. Adolescents who have tried classic psychedelics were significantly more likely to fall into the following demographic categories: older, male, White, and more likely to engage in risky behavior. There were no significant differences in the income levels of those who had versus had not tried psychedelics.

**Table 1:** Demographic of those who have versus have not used classic psychedelics.

Characteristic	Non-users of classic psychedelics [unweighted *N* + (Weighted %)]	Lifetime classic psychedelic users [unweighted *N* + (Weighted %)]	*p* value^1^
**Age (years)**			< 0.001
12	40,071 (16%)	161 (2.9%)	
13	43,118 (17%)	268 (4.4%)	
14	43,364 (17%)	455 (7.7%)	
15	44,198 (17%)	1002 (16%)	
16	43,945 (17%)	1664 (28%)	
17	41,938 (16%)	2433 (41%)	
**Sex**			< 0.001
Male	130,498 (51%)	3477 (59%)	
Female	126,136 (49%)	2506 (41%)	
**Race**			< 0.001
Non-Hispanic White	146,702 (56%)	4066 (70%)	
Non-Hispanic Black	35,433 (15%)	185 (3.8%)	
Non-Hispanic Native American/Alaska Native	3798 (0.6%)	248 (2.2%)	
Non-Hispanic Native Hawaiian/Pacific Islander	1192 (0.4%)	20 (0.3%)	
Non-Hispanic Asian	8823 (4.7%)	84 (2.0%)	
Non-Hispanic more than one race	11,776 (2.5%)	376 (3.9%)	
Hispanic	48,910 (21%)	1004 (18%)	
**Income**			0.4
< $20,000	44,133 (17%)	1072 (16%)	
$20,000-$49,999	80,166 (30%)	1950 (31%)	
$50,000-$74,999	45,173 (17%)	1077 (17%)	
$75,000 +	87,162 (37%)	1884 (36%)	
**Self-reported engagement in risky behavior**			< 0.001
Never	88,263 (36%)	634 (11%)	
Seldom	86,058 (34%)	1525 (27%)	
Sometimes	67,730 (26%)	2567 (42%)	
Always	12,027 (4.4%)	1233 (20%)	

^1^Chi-squared test with Rao & Scott's second-order correction.

The results of our main models testing the associations between MDMA/ecstasy and classic psychedelics and STBs are presented in Table 2, along with the frequency and prevalence of lifetime use for each substance. Psilocybin conferred significant lowered odds of all three outcome measures: lifetime suicidal thinking (aOR 0.85), planning (aOR 0.78), and attempt (aOR 0.77). On the other hand, LSD conferred increased odds of all three outcomes: lifetime suicidal thinking (aOR 1.20), planning (aOR 1.35), and attempt (aOR 1.23). MDMA/ecstasy, peyote, and mescaline did not share significant associations with any of these outcomes.

**Table 2:** Frequency and prevalence of lifetime MDMA/ecstasy and classic psychedelic use + results from survey-weighted multivariable logistic regression models examining the associations between MDMA/ecstasy and classic psychedelics and lifetime suicidal thinking, planning, and attempt.

		Lifetime suicidal thinking	Lifetime suicidal planning	Lifetime suicide attempt
Lifetime use	Frequency + Prevalence [unweighted *N* + (Weighted %)]	aOR (95% CI)^1^	aOR (95% CI)	aOR (95% CI)
MDMA/Ecstasy	4592 (1.7%)	0.96 (0.84, 1.09)	1.12 (0.96, 1.31)	1.15 (0.96, 1.37)
Psilocybin	4281 (1.4%)	0.84* (0.73, 0.98)	0.78* (0.65, 0.95)	0.77* (0.63, 0.96)
LSD	3158 (1.1%)	1.19* (1.01, 1.41)	1.36** (1.12, 1.64)	1.23* (1.01, 1.51)
Peyote	647 (0.2%)	0.99 (0.68, 1.44)	1.29 (0.84, 1.98)	1.24 (0.79, 1.94)
Mescaline	384 (0.1%)	0.80 (0.53, 1.19)	0.72 (0.45, 1.16)	0.71 (0.42, 1.22)

All aforementioned demographic factors and substance use variables included as covariates

^1^*p < 0.05; **p < 0.01; ***p < 0.001; *aOR* adjusted odds ratio; *CI* confidence interval.

Significant values indicating lowered odds of STBs are in bold.

In Supplemental Table 1, we report the results from robustness analyses that assess whether our associations for psilocybin and LSD remain consistent while additionally controlling for a lifetime major depressive episode (MDE), a major risk factor for suicide. We reconducted our original survey-weighted multivariable logistic regression models while including all of our original independent variables and covariates but also added a lifetime MDE in all models as a covariate as well. When we controlled for a lifetime MDE, psilocybin no longer shared a significant association with lifetime suicidal thinking (aOR changed from 0.84 to 0.86). However, aside from this one difference, our pattern of results remained unchanged (i.e., psilocybin was significantly associated with lower odds of suicidal planning and attempt), demonstrating that our findings for psilocybin and LSD remain stable even when one accounts for a major contributing factor to suicidality.

## Discussion

This study tested the non-causal associations between four commonly used lifetime classic psychedelics (psilocybin, LSD, peyote, LSD) and MDMA with STBs in a large representative sample of U.S adolescents. This study was an extension of Jones and Nock (2022a), which tested the associations of the aforementioned substances to STBs in U.S. adults. Although MDMA/ecstasy was not associated with STBs in this study, lifetime use of psilocybin was associated with significantly lower odds of all three STB outcomes whereas LSD was associated with increased odds of all three outcomes. Importantly, this study yielded a similar pattern of results to Jones and Nock (2022a), which found lifetime psilocybin use to be associated with reduced odds of past month psychological distress and past year suicidal thinking, and LSD to be associated with increased odds of past-year suicidal thinking in adults.

### Limitations

The main limitation to this study is the findings cannot be used to establish a causal relationship between psychedelic use and STBs in adolescents. More specifically, this study cannot establish a causal relationship between psilocybin use and lowered odds of STBs nor between LSD use and increased odds STBs. Future longitudinal research will be needed to more rigorously assess the associations that these substances share with STBs in adolescents. Additionally, because we used binary lifetime use outcomes to measure substance use, we cannot establish the recency nor the frequency of psychedelic use in the study. Future studies that can employ more granular measures of substance use can shed further light on the associations between MDMA/ecstasy, classic psychedelics, and STBs in young people.

Relatedly, the operationalization of the variables in the NSDUH does not allow us to establish temporal precedence between psychedelic use and STBs as well. Since STB outcomes are also assessed as binary variables over participants’ lifetimes, we cannot establish that substance use occurred prior to the onset of STBs. Follow-up work that employs more granular assessments of STBs can support a greater understanding of the impact of psychedelics on STBs in adolescents as well. Next, although we conducted robustness analyses to control for MDEs in our analyses, it nevertheless remains possible that psilocybin lowers odds of STBs by way of alleviating harmful and severe mental health conditions that give rise to STBs. Future studies that can control for a wider range of mental disorders and can assess the clinical severity of participants can overcome this limitation.

Finally, multicollinearity—high levels of intercorrelation between independent variables in a given model—represents a possible limitation to our findings as well. Our models featured multiple psychedelic use variables (MDMA/ecstasy, psilocybin, peyote, mescaline, LSD) and demographic factors that are likely correlated with each other, meaning that multicollinearity was likely present in this study. However, the impact of multicollinearity on our results is likely minimal. The main effect of multicollinearity is to increase the standard errors within a given model, widening confidence intervals and making it less likely for statistical tests to reach significance; however, such an effect is mitigated in large sample sizes like the one included in this study^35^. Thus, although our independent variables may correlate with one another, one can remain confident in the observed findings, particularly given that they align with prior work in this research area^26^.

### Potential harm

Given the association between LSD use and increased odds of STBs in our study, LSD and other classic psychedelics may have caused harm at the individual or group level within our sample. Additionally, since these associations follow a pattern of results from other populations based research on classic psychedelics^26^, future research should be aimed at better understanding the link between naturalistic psychedelic use and adverse outcomes in both adolescents and adults. The need to study the potential harm of psychedelics is especially relevant as these substances are increasingly explored as potential treatments within clinical research. Although future research is needed, there are known risks associated with psychedelic use that may lead to increased odds of STBs.

First, a risk associated with psychedelic use is that adolescent substance use is linked to the onset of substance use disorders, a key risk factor for suicide. For instance, epidemiological research has illustrated that individuals who start drinking at 11–12 years of age have nearly double the rate of problematic alcohol use than do individuals who begin drinking at age 21^36^. Generally, young people have higher rates of substance abuse than those who are older as well^37^. Hence, adolescent psychedelic users may be at increased risk for problematic use of these substances and ultimately may be at higher risk for STBs. Future research should examine whether earlier psychedelic use is associated with increased risk of substance use disorders and STBs.

Second, another risk associated with acute classic psychedelic use is a “bad trip” which is characterized by intense fear, anxiety, and/or paranoia while using these substances^38^. The distress experienced in “bad trips” may increase the odds of STBs given how anxiety and paranoia are both well-known risk factors for STBs^9,39^. Furthermore, adolescents might be less equipped to manage the distress engendered from bad trips, as research has demonstrated that younger individuals may be less successful at regulating their emotions^40^. Thus, adolescents may be at particular risk for STBs as a result of adverse psychedelic experiences. Additional investigations that explore the effects of ”bad trips” on adolescent mental health can facilitate greater understanding of our observed findings.

Psychedelic use is also reported to increase the risk of psychosis, although evidence on this link is equivocal^38^. Some historical studies have suggested that psychedelics engender an earlier onset of a psychotic break in individuals with premorbid mental illness^41^. Given that episodes of psychosis are a risk factor for STBs^42^, classic psychedelics could thereby also increase the odds of STBs in adolescents by increasing the risk of an early psychotic episode^43^.

### Potential mechanisms

Our study also presents non-causal associations between psilocybin and lowered odds of STBs in adolescents that builds on previous work by Jones and Nock (2022a) linking psilocybin use with reduced odds of past year suicidal thinking in adults. Several mechanisms may underlie our findings linking psilocybin use to lowered odds of STBs in adolescents, although the below mechanisms remain speculative.

#### Third variable factors

For one, psilocybin use could have been associated with lowered odds of STBs in our sample because of demographic differences between adolescents who have versus have not used psilocybin. Our analysis controlled for confounding demographic variables such as age, sex, race/ethnicity, and yearly household income, but it is plausible that demographic factors not measured in the data might have driven the association between psilocybin use and lowered odds of STBs. Future studies that examine the associations between demographics, LSD use, and STBs can shed light on these potential mechanisms.

Additionally, there may be pre-drug differences between individuals who have versus have not used classic psychedelics, as previous work has found personality differences between psychedelic-using and psychedelic-naive adults that could extend to adolescent populations. Erritzoe et al.^44^ found that psychedelic users score higher on openness ratings and Johnstad^45^ found that psychedelic users score higher on each of the Big Five personality traits except for extraversion (agreeableness, openness, conscientiousness, and neuroticism). Thus, future work should further examine pre-drug differences associated with those who have versus have not used psychedelics, as investigations such as these may further explain our observed associations between psychedelics and STBs.

Finally, there may be clinically-relevant third variable factors that also underlie these findings as well. For instance, there is robust evidence indicating that perceived social support from peers and school teachers is associated with STBs in adolescents, with greater support being linked to lowered odds of STBs^46,47^. If such differences exist within adolescent populations who have used psilocybin—such that adolescents who have used psilocybin spend more time with supportive peers or go to school in more supportive environments—these clinically relevant third variable factors may also underlie our observed findings. Better understanding the relationship between such factors and psilocybin use may further clarify the link between psilocybin and lowered odds of adolescent STBs.

#### Pharmacological pathways

There are potential pharmacological explanations for our observed findings, although these mechanisms remain purely speculative. For instance, psilocybin may lower odds of STBs via interacting with and downregulating serotoninergic 5-HT2A receptors^48,49^. Suicidal behavior has been linked to alterations in the serotonergic system^50,51^ and the activation of 5-HT2A receptors has been found to reduce markers of inflammation in adults that are linked to STBs and related mental health disorders^52^. Additionally, many mainline antidepressants and antipsychotics potentiate their effects through 5-HT2A receptors as well^53,54^. Thus, it is plausible that psilocybin could have lowered odds of STBs among our sample population through interactions with 5-HT2A receptors^50,55^. However, as none of the pharmacological mechanisms of psilocybin have been explored in adolescents, future pharmacological work is needed to better understand the link between psychedelic use and STBs in young people. These investigations can also further elucidate the mechanisms underlying any potential harm that may be associated with adolescent psychedelic use as well.

## Conclusion

Although classic psychedelic use among adolescents has been rising, and more attention has been given to the therapeutic and clinical potential of classic psychedelics in adults, we still know very little about how these drugs affect adolescent mental health. Extending Jones and Nock’s (2022) study on adults, this study similarly found psilocybin to confer with lowered odds of STBs and LSD to confer increased odds of STBs. This study represents an important step forward to better understanding the relationship of psychedelic use to STBs in adolescents and highlights the importance of future cross-sectional, longitudinal, and clinical investigations within this research area.

## Supplementary Information

Supplementary Information.

## Data Availability

The data from this project are publicly available at the Substance Abuse & Mental Health Data Archive (SAMHDA) at the following address: https://www.datafiles.samhsa.gov/.
