# Supplementary material for: Associations between MDMA/ecstasy, classic psychedelics, and suicidal thoughts and behaviors in a sample of U.S. adolescents
Source: Sci Rep. 2022 Dec 19;12:21927. doi: 10.1038/s41598-022-25658-5 (PMC9763256; doi:10.1038/s41598-022-25658-5)
Supplement: Supplementary file 1 — Supplementary Information. [file 41598_2022_25658_MOESM1_ESM.docx]

**Supplemental Table 1.** Results from survey-weighted multivariable logistic regression models examining the associations between psilocybin and LSD and lifetime suicidal thinking, planning, and attempt. **Lifetime MDE is included as a covariate in these models,** in addition to all aforementioned independent variables and covariates.

|  | Lifetime Suicidal Thinking | Lifetime Suicidal Planning | Lifetime Suicide Attempt |
| --- | --- | --- | --- |
| Lifetime Use | aOR (95% CI)^1^ | aOR (95% CI) | aOR (95% CI) |
| Psilocybin | 0.86 (0.70, 1.06) | **0.78* (0.63, 0.97)** | **0.78* (0.62, 0.97)** |
| LSD | 1.44** (1.12, 1.87) | 1.50*** (1.21, 1.87) | 1.27* (1.00, 1.61) |
| ^1^*p<0.05; **p<0.01; ***p<0.001; aOR = adjusted odds ratio; CI = confidence interval | | | |
|  | | | |
